# Social Frailty and Health-Related Quality of Life in Community-Dwelling Older Adults

**DOI:** 10.3390/ijerph19095659

**Published:** 2022-05-06

**Authors:** Young Ko, Kyounga Lee

**Affiliations:** College of Nursing, Gachon University, Incheon 21936, Korea; moodory@gmail.com

**Keywords:** frailty, social factors, quality of life, older adults, community

## Abstract

This study aimed to identify the association between social frailty and health-related quality of life (HRQoL) and to identify the factors influencing HRQoL in community-dwelling older adults in South Korea. This was a secondary analysis of a cross-sectional survey study of 735 older adults. HRQoL was measured using the EuroQoL-5 Dimension, and social frailty was measured using five items. The differences in HRQoL according to sociodemographic characteristics, health-related characteristics, and social frailty of subjects were tested using the Mann–Whitney test, Kruskal–Wallis test, and χ^2^ test. A Tobit regression model was used to identify the influencing factor of HRQoL. About 31.0% of the older adults were in a social pre-frailty state, and 48.4% were in a social frailty state. Of the five components of social frailty, going out less compared to the previous year and being alone were frequently observed. Older adults who had social frailty had lower HRQoL scores than those who were robust after controlling for sociodemographic and health-related characteristics (B_T_ = −0.04, *p* < 0.001). Researchers need to consider older adults’ social frailty as well as health status and sociodemographic status in future interventions to improve the HRQoL of older adults.

## 1. Introduction

The health-related quality of life (HRQoL) is a multidimensional concept that reflects an individual’s subjective attitude and experience with physical, mental, and social health [1]. HRQoL is widely used as an index of health-related outcomes as it is a useful measure of the needs of older adults and their health or wellbeing and, thus, presents information on the effectiveness of relevant interventions and policies [2]. With the increasing older adult population, HRQoL of older adults has become an important factor in the development of national health promotion as well as the establishment and evaluation of public health policies [3].

Various factors, including physical, mental, and cognitive factors, can influence the HRQoL of older adults. Many researchers have recognized the importance of these factors and are employing a variety of strategies to reduce physical, mental, cognitive, and social frailty in community-dwelling or institutionalized older adults. Nowadays, not only traditional interventions, but also interventions using technologies such as game consoles, robots, and virtual reality have a positive effect on the preventing and maintaining frailty of older adults [4,5,6].

Recently, several studies have shown that social factors influence the HRQoL [7,8]. Social factors such as social activities, social support, perceived loneliness, social networks, and living alone substantially influence the health of older adults [9,10]. While many indices are used to assess such social functions and interactions, social frailty is a concept that comprehensively embraces them. Social frailty reflects a spectrum of increased risk or having lost resources required to meet one or more basic social needs during the life span [10]. Until recently, several tools have been developed to measure social frailty [9,11,12]. Among them, the tool developed by Makizako and colleagues [11] was defined as social frailty using five items such as going out less frequently, rarely visiting friends, feeling unhelpful to friends or family, being alone, and not conversing with someone every day. This tool has been used in previous many studies [8,13,14,15,16] because the validity of this tool was confirmed as a strong and independent predictor of disability among older adults living in the community [11]. Social frailty is of particular interest as it relates to the ongoing coronavirus disease 19 (COVID-19) pandemic. Pandemic control measures have increased the social frailty of healthy older adults due to reduced social activity and exchange and lack of social resources [17], and these changes in the context of the COVID-19 pandemic have exacerbated the health status of older adults [18,19]. The concept of frailty includes physical, psychological, cognitive, and social aspects and frailty as a reversible state [13]. Thus, appropriate interventions to prevent social frailty in situations such as the COVID-19 pandemic can help to maintain and ameliorate health status and functions to prevent irreversible states such as morbidity or mortality [20].

Social frailty is important because it influences health outcomes through health behaviors and lifestyles. Social frailty cannot be screened or treated simply with medications but rather requires comprehensive attention to the social environment; thus, it is necessary to pay attention to social frailty in older adults and actively manage consequent problems and implement relevant interventions [21]. A longitudinal study on non-physically frail older adults reported that socially frail older adults were more vulnerable to physical frailty, which, in turn, could lead to social frailty [13]. Moreover, social frailty has been associated with several health-related problems [7,10,22]. Identifying and managing social frailty in older adults will contribute to the prevention and improvement of other health outcomes as well as both physical and mental frailty.

Despite the significance of social frailty in community-dwelling older adults, studies on the influence of social frailty on HRQoL, a health-related index, are lacking. A previous study also highlighted the need for additional studies to determine whether social frailty components, such as social isolation and loneliness, are important factors in identifying frailty and health problems in older adults [9]. If social frailty influences the HRQoL of older adults, it should be well managed in order to boost HRQoL. In this context, this study investigated the association between social frailty and HRQoL in older adults (Aim 1) and examined the influence of sociodemographic factors, health-related factors, and social frailty on HRQoL in older adults (Aim 2).

## 2. Materials and Methods

### 2.1. Study Design and Participants

This study was a secondary analysis of data from a previous cross-sectional study that explored the influencing factors of being homebound among older adults living in the community. In the original study, older adults aged 60 years and older who were able to communicate and who gave written consent to participate in the study were included. A total 840 participants were recruited. Of them, 735 who were 65 years of age or older and had no missing data were included in the analysis. In other words, the sample inclusion criteria for this study were the older adults aged 65 years or over living in the community who could communicate and voluntarily consent to this study. To address the Aim 2, we calculated sample size for a Tobit regression model. The coefficient of the Tobit regression model can be estimated with maximum likelihood estimation [23]. The inference on the slope parameters based on maximum likelihood estimation may be inadequate when the sample size is small, say n ≤ 50 [24]. For several models of regression, such as multiple regression, a minimum of 10 to 15 observations per predictor variable will generally allow good estimates [25,26]. We included 10 variables and so we need 100–150. Therefore, the sample size was sufficient for this study.

### 2.2. Measure

#### 2.2.1. Health-Related Quality of Life: EQ-5D

HRQoL was measured using the EuroQoL-5 Dimension (EQ-5D) tool. The EQ-5D instrument has been widely used in the evaluation of health policy programs [2,27]. The EQ-5D consists of five questions that ask about current health status in five dimensions: mobility (*M*), self-care (*SC*), usual activity (*UA*), pain/discomfort (*PD*), and anxiety/depression (*AD*). Participants responded, “no problems (1)”, “some problems (2)”, or “severe problems (3)”, for each question. From the participants’ answers, EQ-5D scores obtained the formula of the Korea Disease Control and Prevention Agency (KDCA) as estimated weights of EQ-5D [27]. According to the Korean EQ-5D measurement standard presented by the KDCA, if all of the five items are “1”, it is considered to be in a perfect health state, and the EQ-5D value is set to 1. If there is a “2” or “3” response, EQ-5D is 1 h, and h is calculated using the weight formula below.

*h* = 0.05 + 0.096 (*M*2) + 0.418 (*M*3) + 0.046 (*SC*2) + 0.136 (*SC*3) + 0.051 (*UA*2) + 0.208 (*UA*3) + 0.037 (*PD*2) + 0.151 (*PD*3) + 0.043 (*AD*2) + 0.158 (*AD*3) + 0.05 (*N*3).

*M*2 indicates a case in which the response to mobility is “2”, and *M*3 indicates a case in which the response to mobility is “3”. Other items are defined in the same way. The last N3 means that there is a response of “3”. If each item is applicable, 1 is substituted; otherwise, 0 is substituted in the formula. For example, if you answered “3” to all 5 questions, EQ-5D would be −0.171. EQ-5D index has a range from −0.171 to 1, with 1 indicating a perfect health state. The worse the health, the smaller the EQ-5D value [27].

#### 2.2.2. Social Frailty

Social frailty was measured using the tool developed by Makizako and colleagues [11]. This tool consists of five items: being alone (“yes”), going out less frequently compared to last year (“yes”), conversing with someone every day (“no”), visiting the homes of friends sometimes (“no”), and feeling useful to your family and friends (“no”). Each item was answerable by yes or no. If two or more items were responded as vulnerable, the person was classified as “frail”; if there was one item responded as vulnerable, the person was classified as “pre-fail”; and if there was no vulnerable item, the person was classified as “robust”. Social frailty was used as a categorical variable (robust, pre-frail, and frail) in this study. Their validity was supported by previous studies [11,13,14,15,16].

#### 2.2.3. Covariates

Sociodemographic characteristics included sex, age, health insurance type, and education. Age was divided into 65–74 years old, 75–84 years old, and 85 years old and older groups, and was used as a categorical variable. Health insurance was classified into “National Health Insurance” and “Medicaid”. Since “Medicaid” is provided to low-income Koreans, the health insurance type indicates their economic level. Education level was divided into “No education” (0–5 years), “Elementary school” graduate (6–8 years), and “More than middle school” graduate (9 years or more) and used as a categorical variable.

Health-related characteristics included were Instrumental Activities of Daily Living (IADL), the number of chronic diseases, cognitive function, depression, and self-rated health. The IADL indicates the higher level of functioning necessary for independent living. The Korean Instrumental Activities of Daily Living (K-IADL) tool consisting of a total of 10 items was used [28]. Each item was measured as complete independence, partial dependence, and complete dependence. IADL dependency was used as a categorical variable by dividing it into yes and no. In this study, participants were classified IADL dependency as “No” if all items were complete independence, and IADL dependency as “Yes” if they were partial or complete dependence in one or more items.

The number of chronic diseases was calculated as the sum of the number of diagnosed diseases by a doctor including hypertension, diabetes, stroke, cancer, arthritis, urinary incontinence, heart disease, and chronic respiratory disease. Chronic diseases were classified as none, 1–2, or 3 or more, and were used as categorical data.

For cognitive function, the Korean version of Mini-Mental Status Examination for Dementia Screening (MMSE-DS), which is used in Korea public health centers, was used [29]. It consists of a total of 19 items including time orientation, place orientation, memory registration and recall, attention and calculation, command execution, naming, repetition, copying interlocking pentagon, and judgment. It ranges from 0 to 30, with higher scores indicating better cognitive function. According to the cognitive decline criteria considering sex, age, and education level, participants were classified into “Normal” and “Impairment”.

Depression was assessed by using the Geriatric Depression Scale Short Form Korea Version (GDSSF-K), which consists of a total of 15 questions on a 2-point scale [30]. The total score ranged from a minimum of 0 to a maximum of 15, with 0 to 4 being “Normal”, 5 to 9 being “Mild depression”, and 10 to 15 being “Severe depression”.

Self-rated health queries “how do you think your health is in general” and results indicate very poor, poor, average, good, and very good. Self-rated health was used as categorical variables in this study. Very few respondents answered that they were very poor and very good, so very poor and poor were classified as “Poor”, average classified as “Average”, and good and very good as “Good”.

### 2.3. Data Collection

The original study was conducted after approval from the institutional review board of the institution with which the researchers were affiliated (IRB 1044396-201909-HR-174-02). Officers of the public health center of K city informed the older adults living in the community about the study. Participants who wished to participate in this study were recruited through a public health center between September 2019 and November 2020. Data were collected via structured questionnaires by public health officers after obtaining their written agreement.

### 2.4. Statistical Analysis

All analyses were performed using SPSS version 26.0 (IBM Corp., Armonk, NY, USA). The *p*-values < 0.05 were considered to indicate statistical significance.

The score of HRQoL followed a non-normal distribution (Kolomogorov–Smirnov test, *p* < 0.05). We presented both mean with standard deviation and median (range) scores and applied the Mann–Whitney test or Kruskal–Wallis test to identify the significant differences in the summed rank scores of HRQoL. Using the χ^2^ test, we identified the difference between the subdomains of the HRQoL and stage of social frailty. A Tobit regression model was used to determine the influencing factors of the HRQoL score because the HRQoL followed a non-normal distribution and HRQoL was centered at 1.0. Independent variables for which *p*-values were less than 0.05 from the Mann–Whitney test or Kruskal–Wallis test were selected and then included in the model. All independent variables were categorical variables, treated as dummy variables and input to the model. It was confirmed that all variance inflation factors were less than 10, and there was no multicollinearity between independent variables. We conducted a Tobit regression analysis, with social frailty and covariates (sex, age, health insurance type, education, IADL, chronic diseases, cognitive function, depression, and self-rated health) as independent variables and HRQoL as a dependent variable.

## 3. Results

### 3.1. Sociodemographic and Health-Related Characteristic of the Participants and HRQoL

Among the 735 participants, 68.2% were female and 45.6% were 65–74 years old. A total of 79.9% of them were National Health Insurance beneficiaries, and 36.6% graduated from middle school or higher. As for health-related characteristics, 20.3% were IADL dependent, and 25.9% had three or more chronic diseases. In total, 3.8% showed cognitive decline and 8.8% had severe depression symptoms. The number of older adults who answered “Good” about their health was 26.0%. The average HRQoL score was 0.84 (±0.11). There were significant differences in HRQoL according to sex, age, health insurance type, education, IADL dependency, chronic diseases, cognitive function, depression, and self-rated health (*p* < 0.05) (Table 1).

### 3.2. Association of Social Frailty and HRQOL

Table 2 shows the results of analyzing the difference in HRQoL according to the stage of social frailty and its components. About 20.5% were in a robust state, 31.0% were in a social pre-frailty state, and 48.4% were in a social frailty state. A significant difference was noted in the HRQoL according to the stage of social frailty. The HRQoL score followed the order of robust, pre-frailty state, and social frailty state (*p* < 0.001). Among the components of social frailty, the worst was “going out less frequently compared to last year” (52.2%), followed by “being alone” (45.3%), “not visiting the homes of friends sometimes” (32.7%), “not feeling useful to your family and friends” (30.6%), and “not conversing with someone every day” (18.2%). The HRQoL was significantly lower when there was a problem in each component of social frailty (*p* < 0.001).

Table 3 compares scores in the dimensions of the HRQoL according to the stage of social frailty. There was a significant difference between the stage of social frailty and the dimensions of HRQoL (*p* < 0.001). Pain/discomfort (81.7%) was identified as the most problematic among the five dimensions of HRQoL within the social frailty group, followed by mobility (60.1%), anxiety/depression (51.7%), usual activities (44.7%), and self-care (17.1%).

### 3.3. Influencing Factors of HRQOL

Table 4 shows the results of a Tobit regression analysis to determine the factors influencing the HRQoL. The independent variable explained 42.6% of the HRQoL. Social frailty was significantly associated with lower scores of HRQoL (*p* < 0.001). In addition, those who were female, were of an older age, had a low education level, were IADL-dependent, had one or two chronic diseases, suffered from depression, and had average or poor self-rated health were associated with a low HRQoL (*p* < 0.05).

## 4. Discussion

This study investigated the association between social frailty and HRQoL and identified the influencing factors of HRQoL in community-dwelling older adults aged 65 years and over. Regarding the social frailty, the highest number of participants (48.4%) had social frailty, which was followed by pre-frailty stage (31.0%) and robust stage (20.5%). In other words, approximately 80% of the participants had one or more social frailty component, a rate higher than those reported in other countries [8,14,22,31]. A previous study also reported 82.5% of Korean older adults having at least one of these social frailty components [8], which is higher than 50.0% in Japan [14], 53.3% in China [31], and 54.0% in Singapore [22]. This finding suggests a high level of social frailty among Korean older adults, which warrants more attention and management of social frailty in community-dwelling older adults in Korea. HRQoL differs according to the social frailty stage. Older adults with social frailty had the lowest HRQoL scores, and the results of the regression analysis confirmed that the HRQoL score was significantly lower in the social frailty group compared to that in the robust group, highlighting the need for social frailty management in order adults.

Of the components of social frailty, those frequently observed in this study were going out less frequently compared to the previous year and being alone. The association between living alone and a poorer HRQoL has been consistently documented in the literature in many countries [32,33]. In addition, in our study, 52.2% of participants reported going out less compared to the preceding year, with 32.7% and 18.2% indicating that they did not visit their friends’ houses and did not talk to people every day, respectively. In the United Kingdom, 12% of the participants over 65 years of age never spent time with their family or friends, and 17% said they talked to their family, friends, or neighbor less than once per week [34]. These situations increase older adults’ future social isolation, which, in turn, can only cause negative health outcomes such as nutritional, physical, and mental problems that can increase health and social care costs and demand [7]. The recent COVID-19 pandemic has had dramatic effects on several health problems, including mental and physical health, in most of the population, including older adults, due to social isolation and curtailed social interactions [35], which emphasized the importance of managing social frailty. The management of social frailty is essential for overcoming not only individual levels but also public health and community levels [35]. Therefore, a multi-level approach for preventing and managing social frailty can be effective to improve quality of life of older adults.

The HRQoL of older adults differed significantly according to their sociodemographic characteristics, health-related characteristics, and social frailty. HRQoL was noted to be lower in females compared to males, in the older group, in the Medicaid group (lower economic status), and in the uneducated group. These results were similar to previous findings indicating a poorer HRQoL in females, those of an older age group, those in a low economic status, and those with lower education levels [11,36,37]. Regarding health-related characteristics, the HRQoL was lower with more chronic diseases, cognitive decline, depression, and poor self-rated health. These results were consistent with previous findings suggesting that comorbidity was negatively correlated with HRQoL [32,36]. Further, they were in line with the findings of a study that examined 1023 older adults living alone in Korea that identified depression and cognitive function as negative predictors of HRQoL [33]. A review of 74 studies that examined the association between depression and QoL in older adults reported that the severity of depression was associated with poorer QoL repeatedly and stably over time; thus, the authors emphasized the importance of depression management [38].

Older adults with social frailty reported some or severe problems in pain/discomfort (81.7%) and mobility (60.1%) compared to other HRQoL dimensions. These results imply that there is a relationship between social frailty and the problems of mobility and pain/discomfort, which are frequently seen in the older group [36]. Previous studies showed that chronic pain [39] and muscle weakness [40] are independently associated with social frailty supported these findings.

While there are studies regarding the effects of single factors, such as social interactions, social support, and social activities, on HRQoL, few studies have investigated the relationship between social frailty, a comprehensive construct encompassing many social components, and HRQoL, which is one strength of this study. HRQoL is the important health outcome; thus, the influence of social frailty on HRQoL underscore the importance of managing social frailty. Management of social frailty is strategy to support healthy aging. Social frailty is a reversible concept that can be improved through close attention and effort; thus, various interventions targeting social frailty in community-dwelling older adults are needed. In particular, the risk for social frailty is increasing as a result of the unanticipated global disaster of COVID-19. Hence, it is important to recognize the importance of social frailty and manage the condition to enhance the HRQoL and promote healthy aging in community-dwelling older adults.

Frailty is considered a cornerstone in geriatrics because it increases the risk of negative health outcomes in older people [41]. Exercise, nutrition, and cognitive and psychosocial interventions are effective strategies for preventing and managing frailty [42]. There is still a lack of study on social frailty and interventions to prevent or reduce it [10]. Since social frailty is an important factor influencing the HRQoL, various strategies are needed to prevent or reduce social frailty in addition to physical and cognitive frailty in the older adults.

This study had several limitations. First, only older adult residents from K city were enrolled; thus, the findings cannot be generalized to the entire older adult population in Korea. Second, due to its cross-sectional study design, the causality between social frailty and HRQoL cannot be established. To overcome these limitations, longitudinal studies with samples comprising residents of several regions should be conducted in the future. In addition, future longitudinal analyses of the present cohort are needed to further understand determinants of social frailty development and association between quality-of-life domains and the social frailty transitions rates over time.

## 5. Conclusions

We identified the relationship between social frailty and HRQoL, and the influence of social frailty on HRQoL in community-dwelling older adults. The results of this study confirmed that, in addition to the sociodemographic characteristics and health-related characteristics, social frailty is also associated with HRQoL. Furthermore, the presence of two or more social frailty components negatively influenced HRQoL. Thus, various interventions targeting social frailty in community-dwelling older adults are needed. In particular, the risk for social frailty is increasing as a result of the unanticipated global disaster of COVID-19. Hence, it is important to recognize the importance of social frailty and manage the condition to enhance the HRQoL and promote healthy aging in community-dwelling older adults. The findings of this study provide evidence supporting the importance of addressing social frailty in health management.

## Figures and Tables

**Table 1:** Characteristics and health-related quality of life of participants; *N* = 735.

Characteristics	Category	N (%)	HRQoL		*p* ^†^ (Post Hoc Test)
			Mean ± SD	Median (Range)	
Sex	Male	234 (31.8)	0.87 ± 0.09	0.91 (0.51–0.95)	<0.001
	Female	501 (68.2)	0.82 ± 0.11	0.85 (0.36–0.95)	
Age (year)	65–74 ^a^	335 (45.6)	0.87 ± 0.09	0.91 (0.40–0.95)	<0.001
	75–84 ^b^	332 (45.2)	0.82 ± 0.11	0.82 (0.36–0.95)	(a > b > c)
	85+ ^c^	68 (9.3)	0.76 ± 0.11	0.72 (0.38–0.95)	
Health insurance	National health insurance	587 (79.9)	0.85 ± 0.11	0.91 (0.38–0.95)	<0.001
	Medicaid	148 (20.1)	0.80 ± 0.11	0.77 (0.36–0.95)	
Education	No school ^a^	217 (29.5)	0.80 ± 0.11	0.77 (0.36–0.95)	<0.001
	Elementary ^b^	249 (33.9)	0.83 ± 0.10	0.87 (0.51–0.95)	(a > b > c)
	More than middle school ^c^	269 (36.6)	0.88 ± 0.09	0.91 (0.40–0.95)	
IADL dependency	No	586 (79.7)	0.86 ± 0.10	0.91 (0.51–0.95)	<0.001
	Yes	149 (20.3)	0.75 ± 0.11	0.72 (0.36–0.95)	
Chronic disease	None ^a^	70 (9.5)	0.90 ± 0.07	0.91 (0.68–0.95)	<0.001
	1–2 ^b^	475 (64.6)	0.84 ± 0.11	0.87 (0.36–0.95)	(a > b > c)
	3+ ^c^	190 (25.9)	0.81 ± 0.11	0.82 (0.40–0.95)	
Cognitive function	Normal	707 (96.2)	0.84 ± 0.11	0.87 (0.36–0.95)	0.001
	Impairment	28 (3.8)	0.78 ± 0.09	0.77 (0.68–0.95)	
Depression	Normal ^a^	485 (66.0)	0.87 ± 0.09	0.91 (0.38–0.95)	<0.001
	Mild ^b^	185 (25.2)	0.78 ± 0.10	0.77 (0.40–0.95)	(a > b,c)
	Severe ^c^	65 (8.8)	0.76 ± 0.11	0.72 (0.36–0.95)	
Self-rated health	Good	191 (26.0)	0.90 ± 0.06	0.91 (0.60–0.95)	<0.001
	Average	303 (41.2)	0.86 ± 0.09	0.91 (0.54–0.95)	(a > b > c)
	Poor	241 (32.8)	0.77 ± 0.11	0.77 (0.36–0.95)	
Total		735 (100.0)	0.84 ± 0.11	0.91 (0.36–0.95)	

Note: HRQoL = health-related quality of life, SD = standard deviation, IADL = instrumental activities of daily living. ^†^ Mann–Whitney test or Kruskal–Wallis test; significant differences in the post hoc test were determined by the Mann–Whitney test.

**Table 2:** Social frailty and health related quality of life; *N* = 735.

Characteristics	Category	N (%)	HRQoL		*p* ^†^ (Post Hoc Test)
			Mean ± SD	Median (Range)	
Social frailty	Robust ^a^	151 (20.5)	0.90 ± 0.07	0.91 (0.60–0.95)	<0.001
	Pre-frail ^b^	228 (31.0)	0.87 ± 0.09	0.91 (0.54–0.95)	(a > b > c)
	Frail ^c^	356 (48.4)	0.80 ± 0.11	0.77 (0.36–0.95)	
Component 1: being alone	No	402 (54.7)	0.86 ± 0.10	0.91 (0.36–0.95)	<0.001
	Yes	333 (45.3)	0.81 ± 0.11	0.82 (0.38–0.95)	
Component 2: going out less frequently compared to last year	No	351 (47.8)	0.86 ± 0.10	0.91 (0.36–0.95)	<0.001
	Yes	384 (52.2)	0.82 ± 0.11	0.82 (0.40–0.95)	
Component 3: conversing with someone every day	Yes	601 (81.8)	0.85 ± 0.10	0.91 (0.36–0.95)	<0.001
	No	134 (18.2)	0.79 ± 0.11	0.77 (0.38–0.95)	
Component 4: visiting the homes of friends sometimes	Yes	495 (67.3)	0.87 ± 0.90	0.91 (0.54–0.95)	<0.001
	No	240 (32.7)	0.79 ± 0.11	0.77 (0.36–0.95)	
Component 5: feeling useful to your family and friends	Yes	510 (69.4)	0.87 ± 0.10	0.91 (0.40–0.95)	<0.001
	No	225 (30.6)	0.78 ± 0.11	0.79 (0.36–0.95)	

Note: HRQoL = health-related quality of life, SD = standard deviation. ^†^ Mann–Whitney test or Kruskal–Wallis test; significant differences in the post hoc test were determined by the Mann–Whitney test.

**Table 3:** Health-related quality of life dimension and social frailty stage; *N* = 735.

HRQoLDimension	Category	Stages of Social Frailty				χ^2^	*p*
		Robust n (%)	Pre-Frail n (%)	Frail n (%)	Total n (%)		
Mobility	No	126 (83.4)	163 (71.5)	142 (39.9)	431 (58.6)	105.45	<0.001
	Some or severe	25 (16.6)	65 (28.5)	214 (60.1)	304 (41.4)		
Self-care	No	145 (96.0)	203 (89.0)	295 (82.9)	643 (87.5)	17.50	<0.001
	Some or severe	6 (4.0)	25 (11.0)	61 (17.1)	92 (12.5)		
Usual activity	No	130 (86.1)	186 (81.6)	197 (55.3)	513 (69.8)	69.34	<0.001
	Some or severe	21 (13.9)	42 (18.4)	159 (44.7)	222 (30.2)		
Pain/discomfort	No	74 (49.0)	82 (36.0)	65 (18.3)	221 (30.1)	53.14	<0.001
	Some or severe	77 (51.0)	146 (64.0)	291 (81.7)	514 (69.9)		
Anxiety/depression	No	131 (86.8)	155 (68.0)	172 (48.3)	458 (62.3)	71.24	<0.001
	Some or severe	20 (13.2)	73 (32.0)	184 (51.7)	277 (37.7)		

Note: HRQoL = health-related quality of life.

**Table 4:** Influencing factors on the health-related quality of life; *N* = 735.

Characteristics	Category	B_t_	t	*p*
Social frailty	Robust ^†^			
	Pre-frail	−0.01	−1.52	0.130
	Frail	−0.04	−4.32	<0.001
Sex	Male ^†^			
	Female	−0.02	−2.83	0.005
Age	65–74 years ^†^			
	75–84 years	−0.01	−1.07	0.287
	85+	−0.023	−1.91	0.057
Health insurance	National health insurance ^†^			
	Medicaid	−0.01	−0.62	0.539
Education	No school	−0.02	−2.50	0.013
	Elementary	−0.02	−2.66	0.008
	More than middle school^†^			
IADL dependency	No ^†^			
	Yes	−0.06	−6.90	<0.001
Chronic disease	None ^†^			
	1–2	−0.21	−2.03	0.043
	3+	−0.02	−1.94	0.052
Cognitive function	Normal ^†^			
	Impairment	0.03	1.79	0.073
Depression	Normal ^†^			
	Mild	−0.04	−5.10	<0.001
	Severe	−0.42	−3.62	<0.001
Self-rated health	Good ^†^			
	Average	−0.03	−4.24	<0.001
	Poor	−0.08	−8.55	<0.001
Likelihood ratio chi (*p*)		242.43 (<0.001)		

Note: B_T,_ regression coefficients; IADL = instrumental activities of daily living; ^†^ reference.

## Data Availability

The datasets generated and/or analyzed during the current study are not publicly available due to the confidentiality clause contained in the explanatory statement provided to participants. Only researchers with ethics approval can access the data.
